# Health news sharing is reflected in distributed reward-related brain activity

**DOI:** 10.1093/scan/nsaa129

**Published:** 2020-10-16

**Authors:** B P Doré, C Scholz, E C Baek, E B Falk

**Affiliations:** Desautels Faculty of Management, McGill University, H3A 1G5, Montreal, Canada; Amsterdam School of Communication Research, University of Amsterdam, 1001 NG, Amsterdam, Netherlands; Department of Psychology, UCLA, 90095, Los Angeles, USA; Annenberg School for Communication, University of Pennsylvania, 19104, Philadelphia, USA; Department of Psychology, University of Pennsylvania, 19104, Philadelphia, USA; Marketing Department, University of Pennsylvania, 19104, Philadelphia, USA

**Keywords:** health neuroscience, valuation, information propagation

## Abstract

Neuroimaging has identified individual brain regions, but not yet whole-brain patterns, that correlate with the population impact of health messaging. We used neuroimaging to measure whole-brain responses to health news articles across two studies. Beyond activity in core reward value-related regions (ventral striatum, ventromedial prefrontal cortex), our approach leveraged whole-brain responses to each article, quantifying expression of a distributed pattern meta-analytically associated with reward valuation. The results indicated that expression of this whole-brain pattern was associated with population-level sharing of these articles beyond previously identified brain regions and self-report variables. Further, the efficacy of the meta-analytic pattern was not reducible to patterns within core reward value-related regions but rather depended on larger-scale patterns. Overall, this work shows that a reward-related pattern of whole-brain activity is related to health information sharing, advancing neuroscience models of the mechanisms underlying the spread of health information through a population.

Information that diffuses widely in the media environment can influence the behavior of individuals and shape broader directions of societal change. Previous studies have shown that information in targeted brain regions is associated with this diffusion of information (Scholz *et al.*, 2017). However, by focusing on these targeted regions, the approach in previous work has discarded information from the majority of the brain. Here, we sought to build a neural model of health information diffusion, focusing on whole-brain representations of reward value elicited in response to health-related news articles. Beyond enriching our scientific understanding of how and why health information spreads throughout a population, models of this kind may ultimately be able to forecast and enhance the impact of health communication at population scale.

Neuroimaging provides a non-invasive way of monitoring the mechanisms that underlie how people perceive and evaluate stimuli, including messages (like health news articles), with the potential to shape the thoughts and behaviors of a large population of people. In particular, several studies have shown that responses within regions of the brain associated with reward valuation, including the ventral striatum and ventromedial prefrontal cortex (vmPFC) (Bartra *et al.*, 2013), are associated with the population-level impact of diverse stimuli, including music (Berns and Moore, 2012), health campaigns (Falk *et al.*, 2012, 2016), microloan appeals (Genevsky and Knutson, 2015), advertisements (Venkatraman *et al.*, 2015) and health news articles (Scholz *et al.*, 2017). More broadly, neural representations of subjective value and related constructs like identity and self-concept are theorized to be key to behavior change (Berkman *et al.*, 2017; DeStasio *et al.*, 2019). Most existing studies have found that considering information from small regions of the brain explains significant variance in out-of-sample message effects. However, with region-centric approaches, much of the relevant information about message processing distributed across the brain data has not been leveraged, despite the fact that a key strength of functional magnetic resonance imaging (fMRI) is whole-brain coverage.

Drawing from work demonstrating that representations of reward value can be decoded on the basis of activity throughout the brain, recent neurobiological models posit that the functional neuroanatomy of reward value extends beyond the core striatal and vmPFC regions examined in previous studies (Schultz, 2010, 2015; Vickery *et al.*, 2011). In particular, these models propose that diverse brain systems interact to rapidly propagate reward-related information throughout the brain and contribute to value signals, generating a distributed value representation that directs cognition and behavior in a multi-faceted manner (Schultz, 2015). However, it is unclear to what extent distributed brain representations of reward value are related to the population-level impact of information, and whether information distributed across the brain can provide additional information beyond core regions of the reward value system such as ventral striatum and vmPFC.

We sought to address this gap in knowledge with two neuroimaging studies that quantified functional brain responses to New York Times health news articles and estimated relationships between these brain responses and sharing of these articles in the broader population of readers. In particular, we constructed models of these data to address two specific questions. First, does expression of a meta-analytically defined whole-brain reward valuation-related pattern relate to population-level information sharing? Second, does expression of this pattern relate to population-level information sharing beyond what activity within value-related brain regions and self-reports of information value can tell us?

## Method

### Participants

Participants were recruited via an online screening survey for two studies (referred to here as Study 1 and Study 2). Across both studies, to be eligible for the fMRI session, screened participants had to meet standard fMRI eligibility criteria including no metal in the body, no history of psychiatric or neurological disorders, not currently pregnant or breast-feeding and not currently taking psychiatric or illicit drugs. All participants were right-handed. In Study 1, 43 participants were scanned, and in Study 2, 40 participants were scanned. Data from 6 participants were lost due to head motion and/or stimulus presentation errors, leaving a final sample of 39 (28 female, 18–24 years old) participants in Study 1 and 38 in Study 2 (28 female, 18–24 years old). No participants from Study 1 participated in Study 2. The data used here have been reported on in previous papers that focused on activity within core regions of interest associated with reward valuation, social, and self-related cognition (Scholz *et al.*, 2017; Baek *et al.*, 2017; Doré *et al.*, 2019). The current investigation reports novel analyses focused on distributed whole-brain patterns associated with reward valuation.

### Scanner article-viewing task

**Fig. 1.**
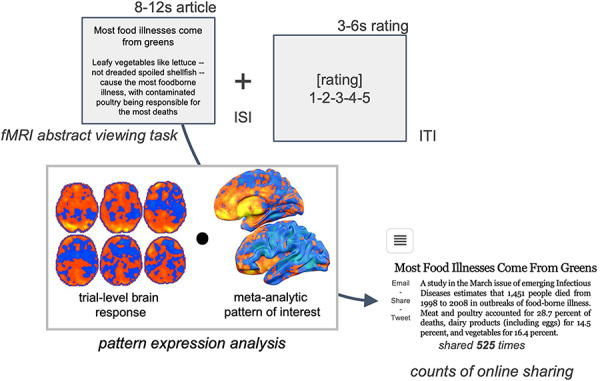
**In-scanner New York Times article viewing task and population information sharing.** Brain activity was collected during participant viewing of headlines and abstracts of New York Times articles focusing on health and fitness. Population-level counts of the number of times each article was shared online within the first 30 days after publication (via email or social media) were collected from the New York Times website. Pattern expression analyses quantified the extent to which each trial-level brain response to a particular article expressed (i.e. showed similarity to) a meta-analytically defined pattern of interest.

As described in previous papers (Scholz *et al.*, 2017; Baek *et al.*, 2017), in both Study 1 and Study 2, participants completed an in-scanner task in which they viewed summaries of news articles (headlines and abstract) from the Health section of the New York Times website (www.nytimes.com) (see Figure 1). The articles were chosen from a census of articles (N = 760) published online in the 7.5 months between 11 July 2012 and 28 February 2013 (Kim, 2015). Articles for the viewing task were chosen from this broader census to maximize comparability in content (i.e. healthy living and physical activity) and length (i.e. word count of title and abstract). Paralleling past reports on Study 1 data, our analyses focused on trials from the scanner task during which participants were asked to consider whether they would read the full text of the article on the basis of the headline and abstract and, at the end of the trial, to indicate (1: very unlikely to 5: very likely) whether they were likely to read the full article (in Study 1). The 80 articles from Study 1 were a subset of those used in Study 2. After reading the headlines and abstracts in Study 2, participants were asked to rate their likelihood to share the article with a specific friend online and their likelihood to post to all of their social media followers. These reading and sharing intention ratings served as our self-report ratings—we treated them separately in models that were fit separately to each study’s data and combined them into a single variable in models that were fit to all of the data from both studies. Although reports of reading intentions and sharing intentions likely draw on only partially overlapping sets of psychological processes, in our data, they are highly correlated (see Supplemental Materials), and as such, in the models that combine them, we treat them as analogous forms of self-reported intentions in the service of fitting models that leverage all of the available data. Population-level data on the number of shares (via e-mail, Twitter and Facebook) that each article achieved in online New York Times readers within 30 days of publication were collected via the NYTimes Application Program Interface (Kim, 2015).

### MRI image acquisition

Neuroimaging data were collected using a 3T Siemens Magnetom Tim Trio scanner equipped with a 32-channel head coil for 40 participants in Study 1 and 33 participants in Study 2, and a Siemens Prisma 3T whole-body MRI with a 64-channel head/neck array was used for one participant in Study 1 and 6 participants in Study 2. Identical specifications were used on both scanners, except for the number of slices acquired for T2*-weighted images (54 at the Tim Trio and 52 at the Prisma scanner; see details in Supplemental Materials). This difference was accounted for in the slice-time correction step during preprocessing. Blood-oxygen-level-dependent signal was captured with a T2*-weighted image sequence [repetition time (TR) = 1.5 s, echo time (TE) = 25 ms, flip angle = 70°, − 30° tilt relative to the anterior commissure–posterior commissure (AC–PC) line, 54 slices at the Magnetom Tim Trio scanner, 52 slices at the Prisma scanner, field of view (FOV) = 200 mm, slice thickness = 3 mm, multiband acceleration factor = 2, voxel size = 3 × 3 × 3 mm]. High-resolution T1-weighted anatomical images were collected using a magnetization-prepared rapid gradient echo (MPRAGE) sequence [inversion time (TI) = 1110 ms, 160 axial slices, voxel size = 0.9 × 0.9 × 1 mm]. Finally, we collected an in-plane, structural, T2-weighted image (slice thickness = 1 mm, 176 axial slices, voxel size = 1 × 1 × 1 mm) to implement a two-stage co-registration procedure between functional and anatomical images.

### fMRI analyses

#### Preprocessing and general linear model fitting.

Data were preprocessed with SPM8, incorporating tools from AFNI and FSL, and consisted of despiking, slice-time correction, realignment, co-registration of functional and structural images and normalization to the standard Montreal Neurological Institute (MNI) brain by segmentation of the structural image. Normalized images were smoothed with an 8-mm Gaussian kernel.

First-level (individual participant) GLM analyses were implemented in SPM8. Analyses used a β-series approach in that each article viewed in the task was modeled as a separate boxcar function convolved with the canonical hemodynamic response, generating separate estimates of brain activity for each article viewing period, for each participant. Six rigid-body motion parameters, and a high-pass filter for 128 s were included as regressors of no interest.

#### Region of interest.

We constructed a region of interest (ROI) analysis in order to extract estimates of brain activity from ventral striatum and vmPFC, identified via meta-analysis as carrying a monotonic, modality-independent signal for subjective reward value (Bartra *et al.*, 2013). Consistent with the notion that these regions tend to activate together as part of a coordinated neural response, their activity was highly correlated from trial to trial (53% shared variance). Therefore, for simplicity, we considered these regions together as single reward value-related ROI (see Supplemental Materials, and also Scholz *et al.*, 2017, for a separate consideration of these two regions; results are substantively similar in both cases).

#### Pattern of interest.

A pattern of interest is a generalization of the concept of an ROI wherein voxels are assigned continuous weights rather than a binary assignment of being included in an ROI or not. To index distributed neural processes related to valuation, we conducted an automated meta-analysis using Neurosynth (Yarkoni *et al.*, 2011). Specifically, we used the Neurosynth core tools (github.com/neurosynth/neurosynth; ver 0.3.7) to conduct an automated meta-analysis identifying brain activity reported more frequently within studies using the term ‘reward’ (922 studies) than within studies not using the term ‘reward’ (13 448 studies). The results of this meta-analysis yielded a pattern wherein the value (z-score) for each voxel reflects the degree to which nearby brain activity is reliably associated in the existing literature with the term ‘reward’ (i.e. an association test or ‘reverse inference’ map). The average activation of the reward value ROI and the multivariate pattern of activity in the reward value meta-analytic pattern variables contain different information in the sense that the ROI variable represents the average activity within a small ROI and the meta-analytic pattern variable represents expression of a continuous pattern of weights throughout the entire brain. Mitigating any concerns of collinearity, these two variables were only weakly correlated in their expression from trial to trial (4% shared variance), indicating that they provide non-redundant information about brain responses.

#### Pattern expression.

We conducted pattern expression analyses to test whether expression of our meta-analytic whole-brain pattern was associated with large-scale information sharing. In order to calculate the extent to which trial-level beta images expressed the pattern of interest, we treated the pattern as a vector of weights and calculated the Pearson correlation between this vector and each vectorized trial-level brain activation image, yielding a correlation coefficient reflecting the similarity between the global brain response and the pattern of interest on each trial for each participant.

#### Sensitivity analysis.

We next examined whether the effects of whole-brain pattern expression on large-scale information sharing were primarily driven by activity within regions of ventral striatum and vmPFC that were present in our ROI analysis. Therefore, we sought to remove clusters from the whole-brain pattern of interest that overlapped with the reward-value ROIs in ventral striatum and vmPFC. In order to identify clusters within the whole-brain pattern that overlapped with the ROIs (so that we could exclude them), we first thresholded the whole-brain meta-analytic pattern by applying whole-brain correction. Specifically, we used a whole-brain Monte Carlo simulation implemented via Alphasim in Neuroelf v 1.1 (neuroelf.net) to threshold this pattern, indicating that clusters comprising at least 50 contiguous voxels at a voxel-wise statistical threshold of z > 2.56 corresponded to a corrected family-wise error rate of <5% under repeated sampling. We then removed any cluster that overlapped with the ventral striatum and vmPFC ROIs. Note that because this voxel-wise threshold is relatively liberal, masking out the identified clusters and re-evaluating the relationship between expression of the masked pattern and population article sharing provides a conservative test of whether the efficacy of the pattern is driven by patterns of activity within and around these core reward value-related brain regions.

### Modeling

We used R (cran.r-project.org; ver 3.4.3), Stan (mc-stan.org; rstan ver 2.18), and the ‘brms’ package (Bayesian Regression Models using Stan ver 2.6.0) to fit hierarchical Bayesian regression models that estimated relationships between our whole-brain pattern of interest, ROI, and self-report predictor variables, and our outcome variable, the log-transform of the number of times each New York Times article was shared (from 34 to 12 740), first within-person (i.e. estimating the relationship between brain activity and article success for the average perceiver), and second the article-to-article relationship between brain activity averages and article success (paralleling results reported by Scholz *et al.*, 2017). Analyses took an estimation approach in that the goal was to generate plausible ranges for population parameters describing relationships between variables within a postulated broader population. Further, we used a Bayesian version of cross-validation to estimate the out-of-sample predictive accuracy of the models, providing a basis for model comparison.

All within-person models incorporated terms allowing coefficients to vary from person to person. Predictor and outcome variables were standardized, yielding as measures of effect size β-coefficients indicating the magnitude of the within-person relationship between the predictor variable and the outcome variable within the typical person. We also fit single-level (i.e. article-level) models by averaging each of our predictor variables (ROI activity, pattern expression, self-reports) by article. In this case, the β-coefficients indicate the magnitude of the relationship between the predictor variable and the outcome variable at the aggregate level of article-to-article variation in average responses. To estimate the variance explained, we used a Bayesian version of R^2^ that entails dividing the model predicted variance by the predicted variance plus the error variance (Gelman and Pardoe, 2006). To estimate the out-of-sample predictive accuracy of our linear models, we approximated Bayesian leave-one-out (LOO) cross-validation using Pareto-smoothed importance sampling (Vehtari *et al.*, 2017). We used this procedure to derive LOO-adjusted deviance value or information criterion (LOOIC) that can be used to compare models in terms of their expected out-of-sample predictive accuracy.

Because weakly informative priors yield inferences that are similar to traditional maximum likelihood estimates but regularize extreme values toward zero, we used weakly informative priors on β-coefficients (overall ‘fixed’ terms for model intercepts and/or slopes), standard deviations (varying ‘random’ terms for intercept and slope variation) and covariances. Specifically, we used a normal prior with location 0 and scale 1 on β-coefficients, a half-normal with location 0 and scale 1 on standard deviations, and an LKJ distribution with regularization parameter 1 on covariances (Lewandowski *et al*., 2009; Stan Development Team, 2016). Models were estimated via Markov Chain Monte Carlo (MCMC) sampling, running four parallel chains for 1000 iterations each (the first 500 samples for each chain were discarded). This number of iterations proved sufficient for convergence in that the Gelman–Rubin diagnostic reached a value of between 0.95 and 1.05 for all parameters (Gelman and Rubin, 1992). In comparison with maximum likelihood based approaches to multilevel modeling, this Bayesian estimation approach offers posterior inference, more accurate estimation of hierarchical variance parameters, better rates of convergence, and diagnostics for assessing the validity of the MCMC-based statistical inferences (Stan Development Team, 2016).

## Results

### Meta-analytic pattern expression is associated with population-level information sharing

#### Within-person models.

**Fig. 2.**
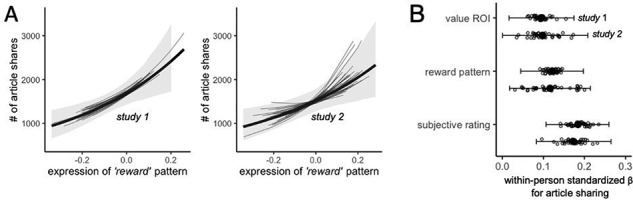
(A) Within person expression of the reward value-related brain pattern was associated with population-level article sharing for both Study 1 (left panel) and Study 2 (right panel) (thick black line with grey band reflects group relationship and 95% CI, thin grey lines reflect person-specific estimates). (B) Coefficient plot visualizing the coefficients for a multilevel model with population article sharing as an outcome variable and activity within the univariate reward value ROI, expression of the reward value pattern of interest, and subjective ratings as simultaneous predictors in a single model (large black dot and interval reflect group coefficients and 95% CI, small grey circles reflect person-specific estimates).

Our initial question was whether expression of the meta-analytically defined reward-value related pattern was related to population-level article sharing (collected via the New York Times website). Within person, the expression of the Neurosynth-defined reward value pattern was associated with population article sharing in both Study 1, β = 0.10, 95% confidence interval (CI)[0.03, 0.18], R^2^ = 0.01, and in Study 2, β = 0.15, 95% CI[0.07, 0.24], R^2^ = 0.02 (see Figure 2). The magnitude of these relationships suggest that the standardized within-person relationship between article-to-article variation in reward value pattern expression and article-to-article variation in (log-transformed) population sharing is about β = 0.10 or β = 0.15 for the typical perceiver. These results indicate that expression of the meta-analytic reward value-related pattern showed a relationship with population-level sharing of the news articles.

#### Between-article models.

From the perspective of practitioners seeking to explain variance in the population-level success of messaging, it is important to characterize our models in terms of how well they explain article-to-article variance in population sharing. Therefore, we also estimated the relationship between the average pattern expression shown to each article (averaged across all the perceivers in a study) and the population sharing of those articles. This indicated that the article-level relationship between pattern expression and population article sharing was β = 0.33, 95% CI[0.12, 0.54], R^2^ = 0.11, in Study 1 and β = 0.41, 95% CI[0.21, 0.62], R^2^ = 0.17, in Study 2.

### Pattern expression relates to information sharing beyond regional brain activity and self-report

#### Within person models.

In a next step, we sought to understand the extent to which a model incorporating expression of the reward value-related pattern explained additional variance in population level sharing, relative to reduced models including only the reward value-related ROI and subjective ratings of the articles. First, we fit models that controlled for activity within the reward value-related ROI (spanning vmPFC and ventral striatum), and self-reports of article value (reading intentions in Study 1, sharing intentions in Study 2), finding that expression of the reward value pattern was associated with population article sharing above and beyond these other variables in both Study 1, β = 0.08, 95% CI[0.01, 0.14], and in Study 2, β = 0.12, 95% CI[0.03, 0.20].

**Fig. 3.**
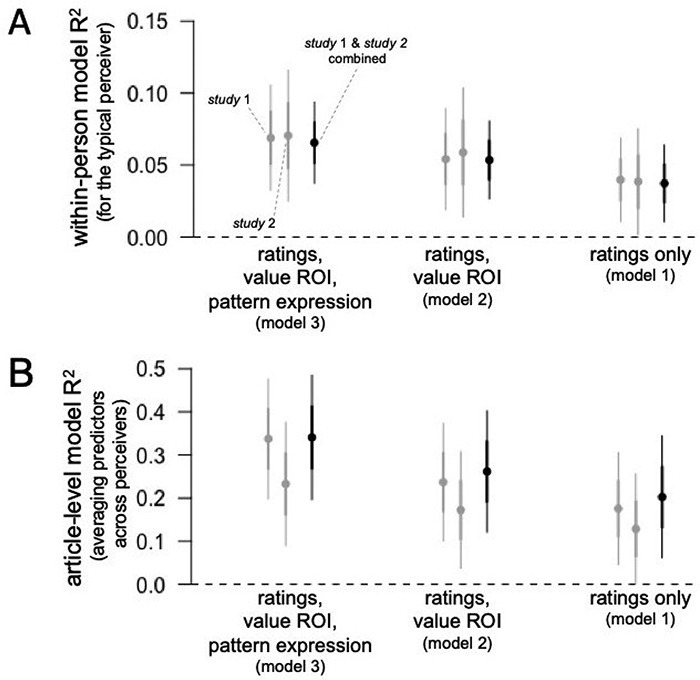
(A) A multilevel model using subjective ratings, reward value ROI activity, and reward value pattern expression from each perceiver could explain about 7% of the variance in population-level article sharing within the typical individual perceiver. (B) An article-level model incorporating ratings, reward value ROI activity and reward value pattern expression averaged across all perceivers could explain about 33% of the variance in population-level article sharing. Error bars represent standard error (i.e. 68% CI) and 95% CI.

Next, we computed R^2^ within multilevel models in order to understand the variance in population article sharing explained by data collected from the typical individual perceiver (see Figure 3A, left panel). Combining the data from Studies 1 and 2, the within-person R^2^ was equal to 0.04, 95% CI[0.02,0.06], for a model 1 including only subjective ratings of the articles, and R^2^ = 0.05, 95% CI[0.03, 0.08], for a model 2 including subjective ratings and reward value-related ROI activity, and R^2^ = 0.07, 95% CI[0.04, 0.09], for a model 3 including subjective ratings, reward value ROI activity, and reward value pattern expression.

We then compared the same models in terms of LOO cross-validated error, in order to evaluate the models in terms of their expected out-of-sample predictive accuracy. The model including self-report ratings, reward value ROI and reward value pattern expression showed substantially improved predictive fit relative to model 1 including only ratings, ∆LOOIC_m3-m1_ = − 29.7, standard error (SE) = 12.0, and relative to model 2 including ratings and reward value ROI activity, ∆LOOIC_m3-m2_ = − 12.9, SE = 7.1. These results indicate that expression of the meta-analytic reward value-related pattern was related to population-level sharing of the news articles above and beyond these region-of-interest and self-report based variables (additional analyses that include different model comparisons support similar conclusions; see Supplemental Materials).

#### Between-article models.

As above, in order to understand the practical value of each predictor in its potential for forecasting out-of-sample sharing, and specifically, to understand the variance explained by our brain and self-report variables when aggregating data from all perceivers up to the article level, we averaged our subjective rating, reward value ROI and reward pattern predictors over all participants into article-level average scores (see Figure 3B, left panel). Here, article-level average expression of the reward value pattern was associated with article-level sharing counts above and beyond the other variables in both Study 1, β = 0.32, 95% CI[0.12, 0.52], and in Study 2, β = 0.26, 95% CI[0.03, 0.49].

Combining the data from Studies 1 and 2, the aggregate article-level R^2^ was 0.18, 95% CI[0.05, 0.30], for a model 1 including only subjective ratings of the articles, R^2^ = 0.24, 95% CI[0.10, 0.36], for a model 2 including subjective ratings and reward value-related ROI activity, and R^2^ = 0.34, 95% CI[0.19, 0.46], for a model 3 including subjective ratings, reward value ROI activity, and reward value pattern expression.

The model including ratings, reward value ROI and reward value pattern expression showed improved predictive fit relative to model 1 including only ratings, ∆LOOIC_m3-m1_ = − 12.7, SE = 7.3, and relative to model 2 including ratings and reward value ROI activity, ∆LOOIC_m2-m1_ = − 8.9, SE = 5.6 (see Figure 3A, right panel). Overall, these results indicate that the expression of the reward value-related pattern substantially improved accuracy in predicting population-level article sharing, with a full model reaching on the order of one-third of the variance explained by our self-report and neuroimaging predictors (additional analyses that include different model comparisons produced similar conclusions; see Supplemental Materials).

### Expression of the meta-analytic pattern reflects distributed activity and is not reducible to patterns of activity within or across reward value-related regions of vmPFC and striatum

The analyses we report above indicated that expression of a pattern meta-analytically defined to index reward value-related processes was related to population article sharing beyond activity in a reward value-related ROI, but they could not address whether the efficacy of the pattern derives primarily because it indexes activity within core vmPFC and ventral striatum regions traditionally associated with reward valuation. Further, they did not address the spatial scale(s) of the information within the pattern that contribute to the relationship with the population-level impact of an article. To address these questions, we constructed versions of the meta-analytic pattern that were systematically modified in order to remove information.

**Fig. 4.**
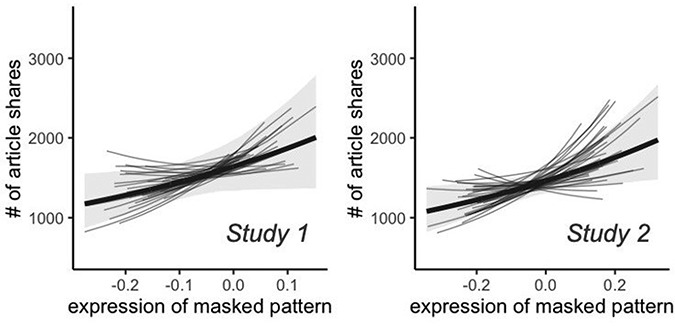
Expression of the meta-analytic map retained a relationship with information sharing when activity within striatum and vmPFC was masked out (removed), indicating that its efficacy is not reducible to patterns of activity within or across vmPFC and striatum.

First, to explore whether the reward pattern was centered primarily on ventral striatum and vmPFC, or a more distributed set of regions, we thresholded the whole-brain reward pattern at a whole-brain family-wise error rate of *P* < 0.05 (a joint cluster-height threshold of z = 2.57 and 50 contiguous clusters). It consisted of both positive clusters (i.e. regions for which activity is reported more frequently in studies that use the term ‘reward’) and negative clusters (i.e. regions for which activity is reported more frequently in studies that do not use the term ‘reward’) distributed widely across the cortex, sub-cortex and brainstem (see Figure 4), and, consistent with past literature, included positive activation within ventral striatum and vmPFC (see Supplementary Table S9 and Supplementary Figure S1). We then created a modified version of the pattern in which clusters that overlapped with our reward value-related ROI (ventral striatum and vmPFC) were masked (removed) from the pattern (that is, all the voxels of the reward value ROI were removed from the pattern as well as any neighboring voxels that were included within clusters of the meta-analytic pattern that overlapped with the reward value ROI). This modified pattern, therefore, included only a subset of the voxels that were included in the original meta-analytically defined pattern. Next, we re-fit our models, finding that expression of this masked pattern retained a within-person relationship with population article sharing in both Study 1, β = 0.11, 95% CI[0.03,0.18], and Study 2, β = 0.12, 95% CI[0.03, 0.20], indicating that the relationship with population level sharing explained by the pattern was not reducible to patterns of activity within reward value-related regions of ventral striatum and vmPFC (see Figure 4). Similarly, the between-article relationship also held for both Study 1, β = 0.28, 95% CI[0.06, 0.49], and Study 2, β = 0.29, 95% CI[0.08, 0.50].

**Fig. 5.**
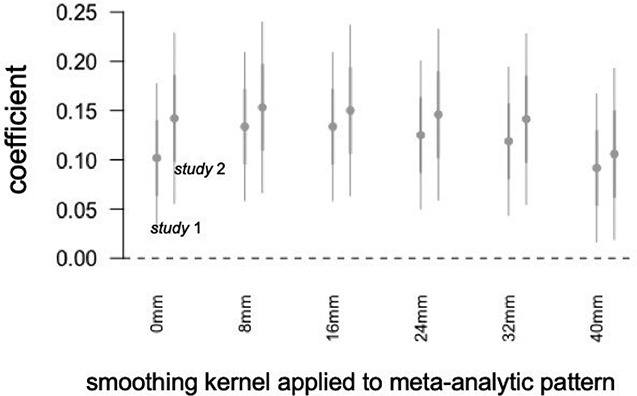
Coefficient plot displaying estimated relationships between expression of the meta-analytic pattern and population article sharing for versions of the reference pattern that were smoothed with a 0- through 40-mm smoothing kernel, while holding constant the smoothness of the participants’ data.

Next, we addressed the question of what spatial scales of information within the meta-analytic pattern contribute to its relationship with information sharing. In order to do so, we created versions of the pattern ranging from unsmoothed to dramatically smoothed (32-mm kernel), fitting models estimating relationships between expression of each of these patterns and population article sharing. The results, summarized in Figure 5, indicated (i) that the relationship with article sharing was numerically highest when the model was smoothed with kernels in the approximate range of 0 to 16 mm and (ii) the model retained clearly non-zero relationships when smoothed with a kernel of 0 to 40 mm. Overall, these results suggest that in addition to targeted regions of interest at relatively local scales, there is also important information at relatively coarse meso- and macro-scales of functional organization, reflecting low spatial frequency (i.e. spread out or spatially coarse) patterns of brain activity that span multiple cortical and subcortical systems.

## Discussion

We used neuroimaging to ask whether whole-brain responses to health-relevant news articles showed relationships with large-scale, out of sample, sharing of those articles, beyond previously identified brain regions of interest and self-report variables. Our results indicated that expression of a distributed pattern of brain activity, meta-analytically associated with reward valuation, was associated with population sharing of the health news articles, beyond previously identified brain and self-report variables. Further, the efficacy of the pattern was not reducible to patterns of activity within core brain reward regions but rather depended on larger-scale patterns of activity distributed widely across cortical, subcortical, and brainstem systems. These findings highlight the advantages of using whole-brain patterns in addition to previously identified region-specific and self-report predictors of health information sharing.

Previous work focused on the brain mechanisms underlying information sharing have shown that core brain regions that track reward value are engaged when disclosing information about the self (Baek *et al.*, 2017; Tamir and Mitchell, 2012), when sharing messages with others (Falk *et al.*, 2012; Baek *et al.*, 2017) and when viewing messages that will be shared most in the population (Scholz *et al.*, 2017; DorÃ© *et al.*, 2019). This work is also consistent with a broader literature that shows that activity in the brain’s reward value system is associated with out of sample outcomes in other domains ranging from music sales (Berns and Moore, 2012) to product sales (Venkatraman *et al.*, 2015) to donation behavior (Genevsky and Knutson, 2015) to health information seeking (Falk *et al.*, 2012, 2016; for reviews see Falk and Scholz, 2018; Knutson and Genevsky, 2018).

Our results build on this work by suggesting a model whereby reward valuation processes that are widely distributed throughout the brain show relationships with population behavior. Further, they indicate that spatially coarse patterns of activity contribute strongly to this relationship, suggesting that it derives from large-scale interactions of distributed brain systems Relatedly, we saw that expression of this value-related pattern was only weakly correlated with average activity within core value-related regions (ventral striatum and vmPFC), and that both the pattern and the region were associated with information sharing. One interpretation of this is that the global pattern and the core regions each index distinct aspects of the brain mechanisms involved in computing the value of sharing the information.

We note that the overall accuracy of the models we present here is still modest in the sense that models including both the value regions and the pattern of interest do not account for a majority of the variance in population sharing. However, addition of the pattern of interest did significantly improve the performance of the model to an appreciable degree—about 4% additional explained variance in article-to-article sharing counts beyond what was explained by self-reports and activity in brain regions of interest alone (max R^2^ = 0.34, including subjective ratings, reward value ROI activity, and reward value pattern expression, and R^2^ = 0.11–0.17 for the reward value pattern on its own, but some variance overlaps between the three main predictors).

These results corroborate the view that focusing on single brain regions in isolation has limits resulting from ignoring large amounts of distributed information across the brain (Kragel *et al.*, 2018; Woo *et al.*, 2017). We provide evidence that representations of the value of information for sharing are widely distributed across the brain. Specifically, although regions identified in the previous literature (ventral striatum and vmPFC) are related to population information sharing, the meta-analytically defined reward pattern was not reducible to these regions. This suggests that brain models will benefit from incorporating distributed whole-brain patterns in order to identify and develop effective signatures of the value and population reach of health-relevant information. Overall, these findings support models of reward valuation in which diverse brain systems interact to rapidly propagate reward-related information throughout the brain (Schultz, 2015), and converge with recent calls for neuroscience models to expand beyond a focus on individual regions in order to incorporate information about distributed patterns and networks (Bassett and Sporns, 2017; Bassett *et al.*, 2018; Kragel *et al.*, 2018). These findings also converge with recent models positing that changes in health behavior, at individual or population scale, may be mediated by changes in neural representations of subjective value and connections to related constructs like identity and self-concept (Berkman *et al.*, 2017; DeStasio *et al.*, 2019). More broadly, they are also consistent with appraisal and constructionist theories positing that emotional and evaluative experiences result from interactions between core affect, sensory, memory, motor, and cognitive systems (Cunningham and Zelazo, 2007; LeDoux, 2012; Lindquist *et al.*, 2012).

The results we report here provide an important step toward identifying valuation-related patterns of brain activity that serve as indicators for components of a population behavioral response to health-relevant information. We propose that patterns of this kind may eventually provide neurobiological measures that usefully supplement self-report evaluations of potentially impactful messages. Further, future work may seek to identify patterns that reflect intermediate psychological and neural processes that link specific kinds of message features to distributed brain representations and in turn to individual- and population-level behavior (i.e. brain pathways that mediate the effects of specific message features on a population behavioral response). Relatedly, testing the specificity and transfer of this whole-brain pattern across different kinds of messages will be key to developing more robust and specific neural signatures of information value and reach.

## Conclusion

If the brain holds information that can be used to forecast large-scale behavior, how do we best characterize and model this activity? Here we suggest that, in addition to summed activity within core value-related brain regions, it is also important for neural models to incorporate information about distributed brain representations of value. In doing so, this work contributes to a mechanistic understanding of how and why information can diffuse (or fail to diffuse) across a population of individuals.

## Supplementary Material

nsaa129_Supp
