# Supplementary material for: Health news sharing is reflected in distributed reward-related brain activity
Source: Soc Cogn Affect Neurosci. 2020 Oct 16;15(10):1111–9. doi: 10.1093/scan/nsaa129 (PMC7657450; doi:10.1093/scan/nsaa129)

**Supplementary Materials**

Health news sharing is reflected in distributed reward-related brain activity

Doré, BP, Scholz, C, Baek, EC, & Falk, EB

**Fitting models that use ventral striatum and vmPFC as separate predictor variables.** Extending analyses reported in Scholz et al. (2018), we fit additional models that used ventral striatum and vmPFC as separate predictor variables of population level news sharing. The two variables (ventral striatum and vmPFC) were highly correlated at both the trial-to-trial level, r = .73, and the article-to-article level, r = .83. In separate bivariate multilevel models, we saw relationships with article sharing at the within-person level for both ventral striatum, β = .08, 95%CI[.06, .11] and vmPFC, β = .09, 95%CI[.06, .12], as well as at the article-to-article level for both ventral striatum, β = .38, 95%CI[.18, .59] and vmPFC, β = .37, 95%CI[.16, .57]. Thus, these coefficients are highly similar. Future work that tests the conditions under which different parts of the reward value system provide similar or distinct information will be informative.

**Additional model R^2^ metrics for models linking pattern expression and population level sharing.** We conducted a series of additional model comparisons to better understand the relative value of the different kinds of predictor variables (self reports, brain regions of interest, brain patterns of interest). First, we estimated R^2^ for the value-related ROI alone for both within-person models, R^2^ = .009 and the between-article models, R^2^ = .13. Second, we estimated R^2^ for the value-related pattern alone for both within-person models, R^2^ = .01, and the between-article models, R^2^ = .16. Third, we estimated R^2^ for the value-related pattern together with ratings for both within-person models, R^2^ = .02, and the between-article models, R^2^ = .23.

**Comparison of bivariate models with only value-related ROI versus only value-related pattern.** In a follow-up analysis, we fit and compared two models with only a single predictor variable – the value-related ROI, and the value-related pattern. In these bivariate models, the value-related ROI was related to population sharing, β = .12, 95%CI[.05, .19], R^2^=.01. The value-related pattern was also related to population sharing, β = .14, 95%CI[.05, .22], R^2^=.02. A comparison of these models indicated that the model using the reward-related pattern was higher in expected out-of-sample predictive accuracy, ∆LOOIC = -1.9, SE = 4.5.

**Fitting models with comparison patterns indexing memory- and vision-related brain processes.** In another follow-up analysis, we fit models that used expression of brain patterns related to processes other than reward in order to ask if these also showed relationships with population article sharing. Specifically, we used meta-analytic maps from Neurosynth for the terms ‘memory’ and ‘vision’. Results from these models indicated that neither expression of the memory-related pattern, β = -.04, 95%CI[-.11, .04], R^2^=.002, or the vision-related pattern, β = .00, 95%CI[-.04, .05], R^2^=.00, was clearly related to population-level article sharing.

**Comparing self-reports of reading intentions and self-reports of sharing intentions.** In our analyses of study 1, we use participants’ self-reports of reading intentions as a self-report rating variable, and in study 2 we use participants’ self-reports of sharing intentions. In these data, we see that both kinds of self-reports show similar relationships with population-level article sharing. For example, at the article-to-article level, we see similar magnitude relationships with population article sharing for both reading intentions in study 1, r = .39, and sharing intentions in study 2, r =.34. Further, since we used the same articles in study 1 and study 2, it is possible to also show that at the article-to-article level, these two self-reports are correlated -- articles that tend to receive high reading intentions also tend to receive high sharing intentions, r = .42. Therefore, despite the fact that these two types of intentions likely reflect some shared and some distinct psychological characteristics, in an effort to make use of all of our data, in models that combine study 1 and study 2, we use both as indices of self-reported article value.

**Table S1.** *One predictor multilevel model in study 1. Using pattern of interest (POI) as a predictor of population sharing.*

Family: gaussian

Links: mu = identity; sigma = identity

Formula: scale(population_sharing) ~ scale(POI) + (scale(POI) | subj)

Samples: 4 chains, each with iter = 1000; warmup = 500; thin = 1;

total post-warmup samples = 2000

Group-Level Effects:

~subj (Number of levels: 39)

Estimate Est.Error l-95% CI u-95% CI Rhat Bulk_ESS Tail_ESS

sd(Intercept) 0.07 0.05 0.00 0.18 1.00 794 1129

sd(scalePOI) 0.10 0.06 0.00 0.24 1.01 589 704

cor(Intercept,scalePOI) 0.04 0.55 -0.94 0.94 1.01 587 967

Population-Level Effects:

Estimate Est.Error l-95% CI u-95% CI Rhat Bulk_ESS Tail_ESS

Intercept 0.01 0.04 -0.08 0.09 1.00 2131 1275

scalePOI 0.10 0.04 0.03 0.18 1.00 1858 1405

Family Specific Parameters:

Estimate Est.Error l-95% CI u-95% CI Rhat Bulk_ESS Tail_ESS

sigma 0.99 0.03 0.94 1.04 1.00 3416 1592

*Notes.* Estimate = mean of the posterior distribution; Est. Error = standard deviation of the posterior distribution; l-95%CI and u-95%CI = lower and upper bound of the 95% credibility interval; Rhat = Gelman-Rubin convergence diagnostic; Bulk_ESS = effective sample size obtained by the MCMC algorithm; Tail ESS = minimum of the effective MCMC sample size at the 5% and 95% quantiles;

**Table S2.** *One predictor multilevel model in study 2. Using pattern of interest (POI) as a predictor of population sharing.*

Family: gaussian

Links: mu = identity; sigma = identity

Formula: scale(population_sharing) ~ scale(POI) + (scale(POI) | subj)

Samples: 4 chains, each with iter = 1000; warmup = 500; thin = 1;

total post-warmup samples = 2000

Group-Level Effects:

~subj (Number of levels: 38)

Estimate Est.Error l-95% CI u-95% CI Rhat Bulk_ESS Tail_ESS

sd(Intercept) 0.06 0.04 0.00 0.15 1.00 570 1104

sd(scalePOI) 0.11 0.05 0.02 0.20 1.01 524 699

cor(Intercept,scalePOI) 0.22 0.52 -0.87 0.96 1.01 321 409

Population-Level Effects:

Estimate Est.Error l-95% CI u-95% CI Rhat Bulk_ESS Tail_ESS

Intercept 0.00 0.03 -0.06 0.06 1.00 1936 1534

scalePOI 0.15 0.04 0.06 0.24 1.00 1593 1128

Family Specific Parameters:

Estimate Est.Error l-95% CI u-95% CI Rhat Bulk_ESS Tail_ESS

sigma 0.99 0.02 0.95 1.02 1.00 2554 1182

**Table S3.** *One predictor article-level model in study 1. Using pattern of interest (POI) as a predictor of population sharing.*

Family: gaussian

Links: mu = identity; sigma = identity

Formula: scale(population_sharing) ~ scale(POI)

Samples: 4 chains, each with iter = 1000; warmup = 500; thin = 1;

total post-warmup samples = 2000

Population-Level Effects:

Estimate Est.Error l-95% CI u-95% CI Rhat Bulk_ESS Tail_ESS

Intercept -0.00 0.10 -0.21 0.19 1.00 1922 1538

scalePOI 0.33 0.10 0.12 0.54 1.00 1753 1229

Family Specific Parameters:

Estimate Est.Error l-95% CI u-95% CI Rhat Bulk_ESS Tail_ESS

sigma 0.93 0.08 0.79 1.10 1.00 1838 1447

**Table S4.** *One predictor article-level model in study 2. Using pattern of interest (POI) as a predictor of population sharing.*

Family: gaussian

Links: mu = identity; sigma = identity

Formula: scale(population_sharing) ~ scale(POI)

Samples: 4 chains, each with iter = 1000; warmup = 500; thin = 1;

total post-warmup samples = 2000

Population-Level Effects:

Estimate Est.Error l-95% CI u-95% CI Rhat Bulk_ESS Tail_ESS

Intercept 0.00 0.11 -0.21 0.21 1.00 1929 1398

scalePOI 0.41 0.10 0.21 0.62 1.00 1867 1205

Family Specific Parameters:

Estimate Est.Error l-95% CI u-95% CI Rhat Bulk_ESS Tail_ESS

sigma 0.96 0.08 0.82 1.13 1.00 1813 1470

**Table S5.** *Three predictor multilevel model in study 1. Using pattern of interest (POI), region of interest (ROI) and rating as predictors of population sharing.*

Family: gaussian

Links: mu = identity; sigma = identity

Formula: scale(population_sharing) ~ scale(rating) + scale(ROI) + scale(POI) + (scale(rating) + scale(ROI) + scale(POI) | subj)

Samples: 4 chains, each with iter = 1000; warmup = 500; thin = 1;

total post-warmup samples = 2000

Group-Level Effects:

~subj (Number of levels: 39)

Estimate Est.Error l-95% CI u-95% CI Rhat Bulk_ESS Tail_ESS

sd(Intercept) 0.06 0.04 0.00 0.14 1.00 805 1031

sd(scalerating) 0.03 0.02 0.00 0.08 1.00 1119 976

sd(scaleROI) 0.03 0.02 0.00 0.09 1.00 1047 780

sd(scalePOI) 0.10 0.05 0.01 0.20 1.00 638 781

cor(Intercept,scalerating) -0.07 0.44 -0.83 0.79 1.00 2405 1319

cor(Intercept,scaleROI) 0.04 0.46 -0.81 0.84 1.00 1838 1394

cor(scalerating,scaleROI) 0.00 0.45 -0.79 0.82 1.00 1519 1474

cor(Intercept,scalePOI) 0.13 0.42 -0.70 0.85 1.00 759 1252

cor(scalerating,scalePOI) -0.11 0.45 -0.87 0.80 1.01 711 1136

cor(scaleROI,scalePOI) 0.02 0.44 -0.78 0.82 1.00 1122 1644

Population-Level Effects:

Estimate Est.Error l-95% CI u-95% CI Rhat Bulk_ESS Tail_ESS

Intercept 0.00 0.03 -0.05 0.06 1.00 2684 1549

scalerating 0.18 0.03 0.13 0.23 1.00 3011 1543

scaleROI 0.07 0.03 0.02 0.13 1.00 2529 1503

scalePOI 0.08 0.03 0.01 0.14 1.00 1787 1272

Family Specific Parameters:

Estimate Est.Error l-95% CI u-95% CI Rhat Bulk_ESS Tail_ESS

sigma 0.97 0.02 0.94 1.01 1.01 2865 1552

**Table S6.** *Three predictor multilevel model in study 2. Using pattern of interest (POI), region of interest (ROI) and rating as predictors of population sharing.*

Family: gaussian

Links: mu = identity; sigma = identity

Formula: scale(population_sharing) ~ scale(rating) + scale(ROI) + scale(POI) + (scale(rating) + scale(ROI) + scale(POI) | subj)

Samples: 4 chains, each with iter = 1000; warmup = 500; thin = 1;

total post-warmup samples = 2000

Group-Level Effects:

~subj (Number of levels: 38)

Estimate Est.Error l-95% CI u-95% CI Rhat Bulk_ESS Tail_ESS

sd(Intercept) 0.09 0.06 0.01 0.21 1.01 852 805

sd(scalerating) 0.07 0.05 0.00 0.18 1.01 744 946

sd(scaleROI) 0.06 0.05 0.00 0.16 1.00 1015 1107

sd(scalePOI) 0.09 0.06 0.00 0.23 1.00 684 1057

cor(Intercept,scalerating) -0.05 0.45 -0.83 0.77 1.00 2732 1415

cor(Intercept,scaleROI) 0.05 0.44 -0.76 0.82 1.00 2696 1675

cor(scalerating,scaleROI) -0.07 0.46 -0.84 0.79 1.00 2032 1400

cor(Intercept,scalePOI) 0.10 0.45 -0.77 0.84 1.00 1657 1551

cor(scalerating,scalePOI) -0.00 0.44 -0.79 0.80 1.00 1724 1548

cor(scaleROI,scalePOI) 0.02 0.45 -0.79 0.82 1.00 1741 1894

Population-Level Effects:

Estimate Est.Error l-95% CI u-95% CI Rhat Bulk_ESS Tail_ESS

Intercept 0.00 0.04 -0.08 0.08 1.00 2581 1740

scalerating 0.19 0.04 0.11 0.27 1.00 3006 1473

scaleROI 0.09 0.04 0.02 0.17 1.00 3503 1512

scalePOI 0.12 0.04 0.03 0.20 1.00 2977 1486

Family Specific Parameters:

Estimate Est.Error l-95% CI u-95% CI Rhat Bulk_ESS Tail_ESS

sigma 0.96 0.03 0.92 1.02 1.00 2838 1226

**Table S7.** *Three predictor article-level model in study 1. Using pattern of interest (POI), region of interest (ROI) and rating as predictors of population sharing.*

Family: gaussian

Links: mu = identity; sigma = identity

Formula: scale(population_sharing) ~ scale(rating) + scale(ROI) + scale(POI)

Samples: 4 chains, each with iter = 1000; warmup = 500; thin = 1;

total post-warmup samples = 2000

Population-Level Effects:

Estimate Est.Error l-95% CI u-95% CI Rhat Bulk_ESS Tail_ESS

Intercept 0.00 0.10 -0.19 0.19 1.00 2399 1430

scalerating 0.30 0.10 0.10 0.51 1.00 2322 1538

scaleROI 0.14 0.10 -0.06 0.34 1.00 2144 1375

scalePOI 0.32 0.10 0.12 0.52 1.00 2237 1582

Family Specific Parameters:

Estimate Est.Error l-95% CI u-95% CI Rhat Bulk_ESS Tail_ESS

sigma 0.86 0.07 0.72 1.02 1.00 2223 1308

**Table S8.** *Three predictor article-level model in study 2. Using pattern of interest (POI), region of interest (ROI) and rating as predictors of population sharing.*

Family: gaussian

Links: mu = identity; sigma = identity

Formula: scale(population_sharing) ~ scale(rating) + scale(ROI) + scale(POI)

Samples: 4 chains, each with iter = 1000; warmup = 500; thin = 1;

total post-warmup samples = 2000

Population-Level Effects:

Estimate Est.Error l-95% CI u-95% CI Rhat Bulk_ESS Tail_ESS

Intercept 0.00 0.10 -0.21 0.21 1.00 2225 1591

scalerating 0.10 0.12 -0.12 0.32 1.00 2088 1542

scaleROI 0.22 0.12 -0.02 0.45 1.00 1608 1557

scalePOI 0.26 0.12 0.03 0.49 1.00 1610 1211

Family Specific Parameters:

Estimate Est.Error l-95% CI u-95% CI Rhat Bulk_ESS Tail_ESS

sigma 0.95 0.08 0.82 1.12 1.00 2040 1284

**Table S9.** *Cluster table of Neurosynth ‘reward’ association test map, thresholded at*  z > 2.56, k=50.

Cluster Table of map: "Neurosynth - Reward, Association Test"

x y z | k | max z | mean z | Talaraich label

-----------------------------------------------------------

13 6 -2 | 4782 | 28.1 | 5.1 | RH Lentiform Nucleus (Lateral Globus Pallidus)

3 -26 30 | 72 | 5.3 | 3.6 | RH Cingulate Gyrus (Brodmann area 23)

-50 13 26 | 457 | -4.6 | -3.1 | LH Inferior Frontal Gyrus (Brodmann area 9)

-50 -46 9 | 273 | -4.2 | -3.0 | LH Superior Temporal Gyrus (Brodmann area 22)

24 -67 46 | 150 | -4.1 | -3.0 | RH Superior Parietal Lobule (Brodmann area 7)

48 -66 9 | 102 | -4.1 | -3.0 | RH Middle Temporal Gyrus (Brodmann area 37)

-45 -63 -1 | 194 | -4.1 | -3.0 | LH Middle Temporal Gyrus (Brodmann area 37)

-24 -59 53 | 214 | -4.1 | -3.0 | LH Precuneus (Brodmann area 7)

61 -27 20 | 72 | -4.0 | -3.0 | RH Postcentral Gyrus (Brodmann area 40)

-8 8 52 | 72 | -3.8 | -3.0 | LH Medial Frontal Gyrus (Brodmann area 6)

62 17 14 | 52 | -3.6 | -3.0 | RH Inferior Frontal Gyrus (Brodmann area 44)

**Table S9.** *Surface rendering of Neurosynth ‘reward’ association test map, thresholded at*  z > 2.56, k=50.


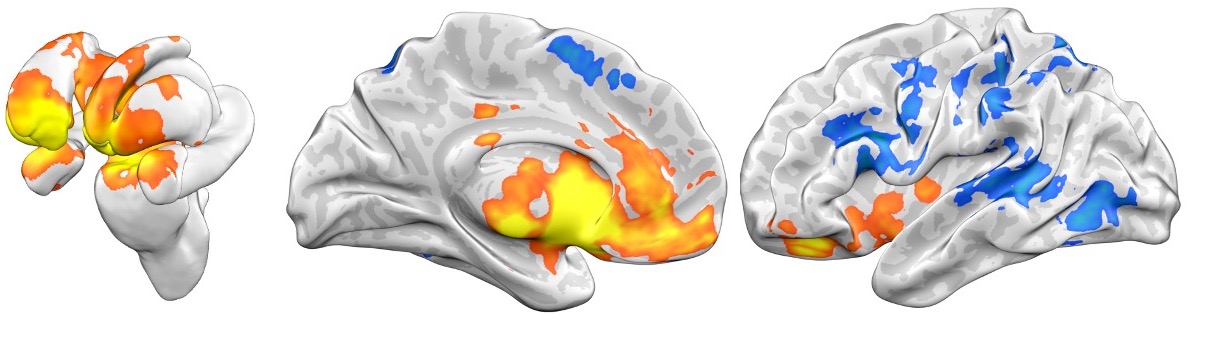

Supplement: nsaa129_Supp [file nsaa129_supp.zip › dore.SCAN.specialissue.supplementarymaterials.aug31.docx]
